# Pangenome analysis of *Lactobacillus mulieris* strains reveals distinct subspecies clusters with defined ecological adaptations

**DOI:** 10.1128/spectrum.02011-25

**Published:** 2025-10-02

**Authors:** Jake Adolf V. Montecillo, Heon Jong Yoo, Yoo-Young Lee, Chul Min Park, Angela Cho, Hyunsu Lee, Hee Yeon Yoon, Jong Mi Kim, Nan Young Lee, Sun-Hyun Park, Nora Jee-Young Park, Hyung Soo Han, Incheol Seo, Gun Oh Chong

**Affiliations:** 1 Department of Immunology, School of Medicine, Kyungpook National University 34986, Daegu, Republic of Korea; 2 Clinical Omics Institute, Kyungpook National University 34986, Daegu, Republic of Korea; 3 Brain Korea 21 FOUR Program, School of Medicine, Kyungpook National University 34986, Daegu, Republic of Korea; 4 Chungnam National University School of Medicine 90159, Daejeon, Republic of Korea; 5 Department of Obstetrics and Gynecology, Gynecologic Cancer Center, Samsung Medical Center, Sungkyunkwan University 198720, Seoul, Republic of Korea; 6 Department of Obstetrics and Gynecology, Jeju National University Hospital, Jeju National University College of Medicine 91577, Jeju, Republic of Korea; 7 Department of Physiology, Pusan National University School of Medicine 540311, Yangsan, Republic of Korea; 8 Department of Obstetrics and Gynecology, Kyungpook National University Chilgok Hospital 34986, Daegu, Republic of Korea; 9 Department of Obstetrics and Gynecology, Kyungpook National University 34986, Daegu, Republic of Korea; 10 Department of Clinical Pathology, School of Medicine, Kyungpook National University 34986, Daegu, Republic of Korea; 11 Division of Advanced Predictive Research, Center for Bio-Signal Research, Korea Institute of Toxicology (KIT) 443298, Daejeon, Republic of Korea; 12 Department of Pathology, Kyungpook National University Chilgok Hospital 34986, Daegu, Republic of Korea; 13 Department of Physiology, School of Medicine, Kyungpook National University 34986, Daegu, Republic of Korea; University of Nevada Reno, Reno, Nevada, USA

**Keywords:** *Lactobacillus mulieris*, subspecies, niche adaptations, pangenome, comparative genomics, phylogenomics

## Abstract

*Lactobacillus mulieris* is a recently described species, reportedly isolated from human urine, vagina, and gut. Previous genomic studies of *L. mulieris* highlighted significant genetic diversity among its strains. To gain a deeper understanding of this genomic diversity, we conducted a comprehensive genomic comparison of 70 *L*. *mulieris* strains from diverse sources. Phylogenomic and genome relatedness analysis identified three distinct clades, each representing a potential subspecies cluster. Pangenome analysis revealed distinct gene clusters shaping the functional characteristics and unique ecological adaptations of each clade. Clade 1 demonstrated a generalist lifestyle, with strains isolated from diverse sources and enriched in serine/threonine protein kinases, suggesting adaptive versatility. Clade 2, predominantly composed of urinary isolates, displayed enrichment in genes facilitating nutrient acquisition and osmotic regulation, enabling survival in the nutrient-limited and high osmolarity conditions of the urinary tract. Clade 3, exclusively composed of vaginal isolates, exhibited significant enrichment in genes supporting glycogen metabolism, carbohydrate transport, and capsular polysaccharide biosynthesis—features indicative of adaptation to the vaginal environment. Collectively, our findings provide essential genomic insights into the ecological specialization of *L. mulieris*, shedding light on their genetic variability and adaptive traits within their respective ecological niches.

**IMPORTANCE:**

Recognizing the genomic diversity within *Lactobacillus mulieris* is essential for understanding its ecological specialization and adaptation strategies across distinct human-associated environments. By identifying three distinct clades with unique functional traits, our study highlights the critical role of niche-specific genetic adaptations in microbial survival. The presence of specialized gene functions within each clade underscores how evolutionary pressures shape bacterial resilience in different environments. Despite their coexistence in overlapping environments, these clades exhibit distinct genomic profiles that may influence their colonization potential and interactions with the host and within the host-associated microbiota. Our findings emphasize the need for a classification framework that accounts for these genetic and functional differences and the necessity for further investigation to understand their distinct roles and impact on human health.

## OBSERVATION

*Lactobacillus mulieris* is a recently described species of *Lactobacillus*, with its type strain, c10Ua161M^T^, isolated from the urine sample of a healthy, reproductive-age woman (1). In addition to urine samples, several strains of *L. mulieris* reported in publicly available repositories have been isolated from diverse sources, including the gut and vagina. While lactobacilli are known to confer beneficial effects (2), the impact of *L. mulieris* on human health and its functional contributions within the microbiota remain largely underexplored. Previous comparative genomic studies of 25 *L*. *mulieris* strains revealed key functional and genotypic differences from its closely related species, *Lactobacillus jensenii* (3, 4). Moreover, these studies identified a distinct subclade within *L. mulieris* strains, suggesting the presence of an emerging genomospecies. In this study, we conducted a comprehensive genomic comparison of *L. mulieris* strains to further expand the existing knowledge of their genomic diversity. Our findings identified distinct clades representing different subspecies clusters, each exhibiting unique adaptations, thereby providing insights into their potential biological functions within their specific ecological niches.

**Fig 1:**
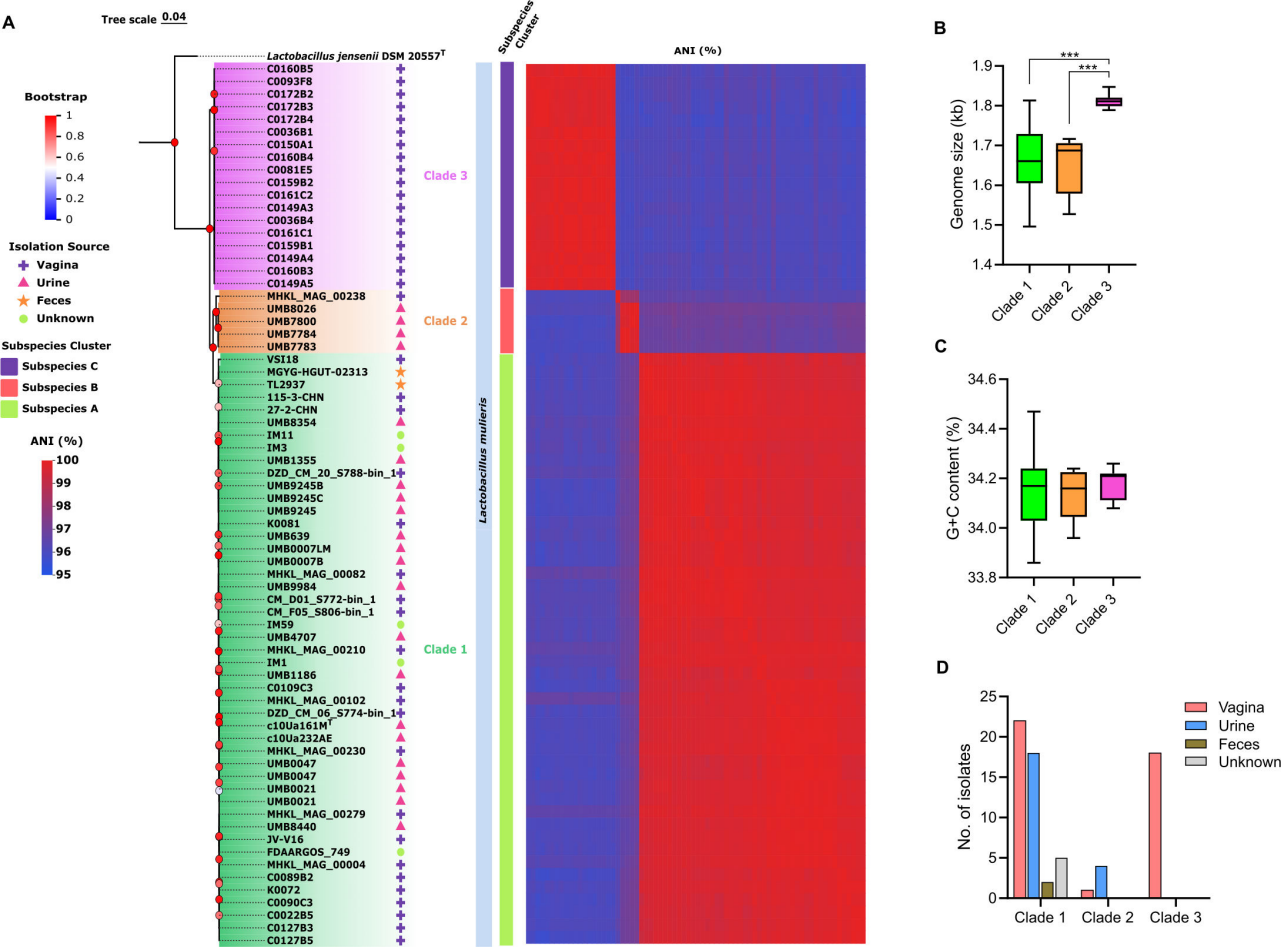
*L. mulieris* is composed of three monophyletic clades, each representing potential subspecies. (**A**) Phylogenomic tree of 70 *L*. *mulieris* strains based on single-copy core genes. Bootstrap support values are indicated near the branching point. Scale bar, 0.04. Distinct clades are indicated in different colors. Pairwise average nucleotide identities (ANIs) are also presented as a heat map. Subspecies clusters inferred through their shared digital DNA-DNA hybridization (dDDH) values are also indicated. (**B**) Genome sizes of strains belonging to each distinct clade. Asterisks represent statistical significance at ***P ≤ 0.001. (**C**) G + C content of strains in each clade. (**D**) Proportion of strains in each clade based on isolation sources.

We analyzed 70 publicly available genome sequences of *L. mulieris* strains (Table S1), encompassing isolates from gut (feces), urine, vagina, and unknown sources. To confirm the identity of these strains, phylogenomic analysis (Supplementary Methods) using single-copy core genes was employed (Fig. 1A). The strains formed a well-supported monophyletic cluster, distinct from *L. jensenii*, supporting their species-level classification. The phylogenomic analysis further delineated the *L. mulieris* strains into three discrete clades. Two of these clades (clades 1 and 2) have been previously reported (3, 4), while clade 3 emerged as a new distinct group. These phylogenomic distinctions were further reinforced by their shared average nucleotide identity (ANI) values. Notably, clade 3 exhibited significantly larger genome size compared with clades 1 and 2 (Fig. 1B), although all three clades showed no significant difference in their G + C content (Fig. 1C). Interestingly, clade 3 exclusively comprised strains of *L. mulieris* that were isolated from the vagina (Fig. 1D), whereas clade 1 represented strains from diverse sources. Clade 2, on the other hand, was predominantly composed of strains isolated from urine samples. Further genomic analysis of these clades using the Type Strain Genome Server (TYGS) (5) revealed their subspecies designation, as reflected by their intra- and inter-clade shared digital DNA-DNA hybridization (dDDH) values (Fig. 1A), corroborating the suggested threshold of 79%–80% dDDH (6). This finding aligns with the previous reports indicating the presence of genomospecies or subspecies of *L. mulieris* (3, 4).

**Fig 2:**
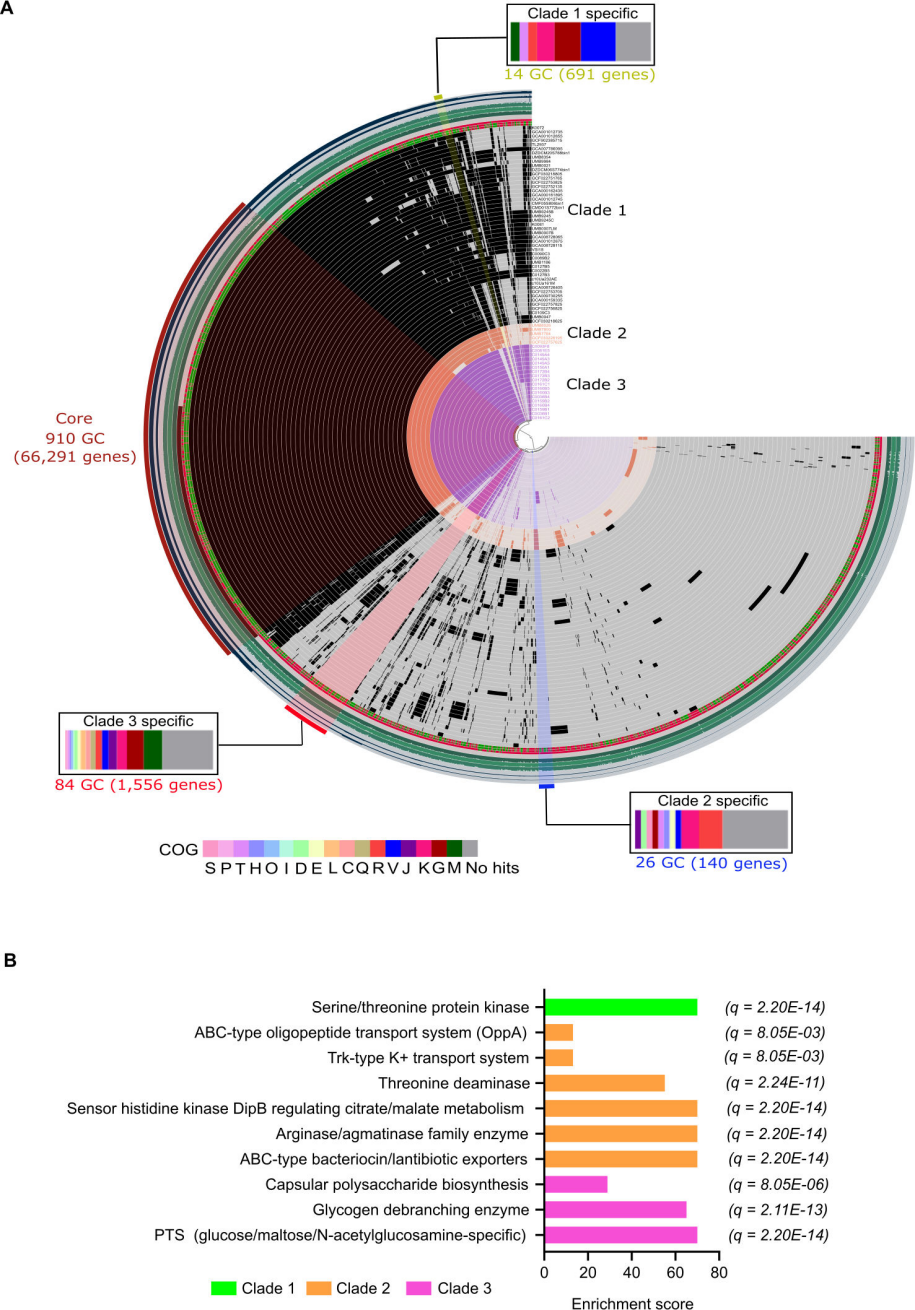
Pangenome of *L. mulieris* unveiled niche-specific adaptations of distinct clades. (**A**) Pangenome of *L. mulieris* showing the consistent clustering of strains into distinct clades based on the presence and absence of orthologous gene clusters (GCs). The core and clade-specific GCs are indicated. Statistics for the pangenome analysis are shown, starting from the outer ring: number of contributing genomes, number of genes in GC, maximum number of paralogs, geometric homogeneity index, functional homogeneity index, combined homogeneity index, single-copy gene clusters (SCGs), and the presence (green) and absence (red) of a clusters of orthologous groups (COG) functional assignments. Functional annotations based on COG category are presented for the GCs unique to each clade. COG S: Function unknown, *P*: Inorganic ion transport and metabolism, T: Signal transduction mechanisms, H: Coenzyme transport and metabolism, O: Posttranslational modification, protein turnover, chaperones, I: Lipid transport and metabolism, D: Cell cycle control, cell division, chromosome partitioning, E: Amino acid transport and metabolism, L: Replication, recombination and repair, C: Energy production and conversion, Q: Secondary metabolites biosynthesis, transport and catabolism, R: General function prediction only, V: Defense mechanisms, J: Translation, ribosomal structure and biogenesis, K: Transcription, G: Carbohydrate transport and metabolism, M: Cell wall/membrane/envelope biogenesis. (**B**) Selected enriched functions identified in each clade. Functional enrichment analysis was conducted using Rao test statistic for equality of proportions, implemented in Anvi’o v.8. To account for multiple testing, P-values were corrected using the false discovery rate (FDR) method, and functions with adjusted *q*-values below 0.05 are presented.

Pangenome analysis using Anvi’o v.8 (7) was then employed to identify gene clusters (GCs) that may shed light on their possible subspecies or clade-specific functionalities. Our analysis revealed clustering of the strains into three distinct clades based on the presence and absence of GCs (Fig. 2A), consistent with their phylogenetic placement (Fig. 1A). Each clade exhibited unique and conserved sets of GCs. The three clades shared 910 core GCs comprising 66,291 genes. Clade-specific GCs were also identified, with clade 3 harboring the highest unique GCs (84), followed by clade 2 with 26 GCs, and clade 1 with 14 clade-specific GCs. Clusters of orthologous groups (COGs) annotation (Data S1) revealed functional specialization among clade-specific GCs. Clade 3-specific GCs showed the highest abundance of genes associated with carbohydrate transport and metabolism (G) and cell wall/membrane/envelope biogenesis (M), suggesting adaptations related to nutrient acquisition and exopolysaccharide production. In contrast, clade 1-specific GCs were enriched with genes functioning in defense mechanisms (V), possibly explaining the high prevalence of prophages in their genomes (Fig. S1). Clade 2-specific GCs were predominantly composed of genes categorized under general function (R). Comparative gene functional enrichment analysis provided further insights into the ecological niches uniquely occupied by each clade (Fig. 2B; Data S2), underscoring their distinct functional adaptations. Clade 1 exhibited significant enrichment in genes encoding serine/threonine protein kinases, which play a crucial role in bacterial signaling, adaptation, and responses to environmental conditions (8). The prevalence of these kinases suggests that clade 1 strains may adopt a generalist lifestyle, consistent with their diverse isolation sources. Clade 2 harbored genes enriched for functions related to adaptation and survival in human urine (9–12). Unlike other nutritionally diverse body sites, urine in the bladder presents a high osmolarity, nutrient-limited environment composed primarily of amino acids and small peptides (9). To persist in this challenging niche, bacteria like the uropathogenic *E. coli* employ strategies for efficient nutrient acquisition and osmotic regulation (10, 11). Among the enriched genes in clade 2, the sensor histidine kinase DipB, which regulates citrate/malate metabolism, likely facilitates metabolic adaptation to available citrate and malic acid in urine (11, 12). Additionally, arginase/agmatinase family enzymes and threonine deaminase contribute to amino acid metabolism (10), providing essential nitrogen sources for survival. The ABC-type oligopeptide transport system (OppA) has been reported to enhance peptide scavenging in urine (9). Furthermore, the Trk-type K^+^ transport system, as previously demonstrated (12), supports osmoregulation, maintaining intracellular homeostasis under high-salt conditions. These genetic features likely enable clade 2 strains to thrive in the urinary tract by optimizing nutrient uptake and physiological resilience. Additionally, clade 2 exhibited enriched ABC-type bacteriocin/lantibiotic exporters. Indeed, clade 2 exclusively possessed enterocin and penocin type of bacteriocins, implying enhanced protective capabilities against microbial competitors. Clade 3 strains appeared well-adapted to the vaginal environment, as evidenced by significantly enriched genes supporting survival in this niche. Glycogen debranching enzymes facilitate glycogen metabolism (13, 14), enabling bacterial utilization of glycogen-derived sugars—a crucial energy source in the vaginal ecosystem (14). The phosphotransferase system (PTS) (glucose/maltose/N-acetylglucosamine-specific) enhances carbohydrate transport and metabolism, particularly from glycogen and mucin degradation (15). The enrichment of genes related to capsular polysaccharide biosynthesis suggests enhanced colonization potential and interactions with the host (16, 17). Together, these enriched genetic features underscore the adaptive mechanisms that enable clade 3 strains to establish and persist within the vaginal environment.

The genomic diversity and ecological specialization observed across the three clades of *L. mulieris* underscore the species’ remarkable adaptability to distinct host-associated niches. In the context of the human microbiome, the functional partitioning observed among clades likely enhances their resilience and fosters host-microbe symbiosis by enabling site-specific metabolic and immunomodulatory interactions (18, 19). Similar to niche-specific adaptations reported in other lactobacilli (20–22), the specialized genetic enrichment across *L. mulieris* clades reflects evolutionary pressures that shape microbial functionality in response to host physiology. These findings support the view that microbial specialization within host niches contributes to ecosystem stability, metabolic complementarity, and immune modulation (18–22), positioning *L. mulieris* as a dynamic and ecologically responsive component of the human microbiome.

Unlike previous works that focused on species-level differentiation between *L. mulieris* and *L. jensenii* (3, 4), our study systematically dissects intraspecies genomic diversity and links functional gene clusters to ecological specialization. Although earlier works have noted the genetic divergence within *L. mulieris* (3, 4), particularly the emergence of a distinct subclade, their analysis lacked functional genetic characterization of these differences and did not explore the possible functional implications of the observed genomic heterogeneity. With the current comprehensive data set of 70 strains, our study advances beyond this limitation by linking genomic diversity to functional adaptation, revealing three distinct and ecologically specialized clades. This finding represents a significant advancement from earlier works by demonstrating that the genomic heterogeneity within *L. mulieris* reflects coherent ecological adaptations rather than stochastic genetic variation.

Collectively, our comparative genomics study delineated distinct clades of *L. mulieris*, representing subspecies with specialized functionalities. The distinct genomic profiles of each clade suggest they may play differentiated roles in host health, potentially influencing site-specific microbial dynamics, immune responses, and disease susceptibility. Future studies integrating multi-omics and functional assays will be essential to elucidate the clinical relevance and therapeutic potentials of these *L. mulieris* clades within the broader landscape of microbiome-host interactions. Formal establishment of these subspecies should be proposed once representative isolates from clades 2 and 3 have been obtained and deposited in public culture collections.

## Data Availability

Data generated and analyzed in this study are provided in this article and its supplemental materials.

## SUPPLEMENTAL MATERIAL

Data S1 spectrum.02011-25-s0001.xlsx Clade-specific gene clusters.

Data S2 spectrum.02011-25-s0002.xlsx Enriched functions.

Figure S1 spectrum.02011-25-s0003.docx Average number of prophages.

Supplemental methods spectrum.02011-25-s0004.docx Supplemental methods and references.

Table S1 spectrum.02011-25-s0005.docx Genome sequence dataset information.

ASM does not own the copyrights to Supplemental Material that may be linked to, or accessed through, an article. The authors have granted ASM a non-exclusive, world-wide license to publish the Supplemental Material files. Please contact the corresponding author directly for reuse.
