# Supplementary material for: Pangenome analysis of Lactobacillus mulieris strains reveals distinct subspecies clusters with defined ecological adaptations
Source: Microbiol Spectr. 2025 Oct 2;13(11):e02011-25. doi: 10.1128/spectrum.02011-25 (PMC12584728; doi:10.1128/spectrum.02011-25)
Supplement: Figure S1 — Average number of prophages. [file spectrum.02011-25-s0003.docx]

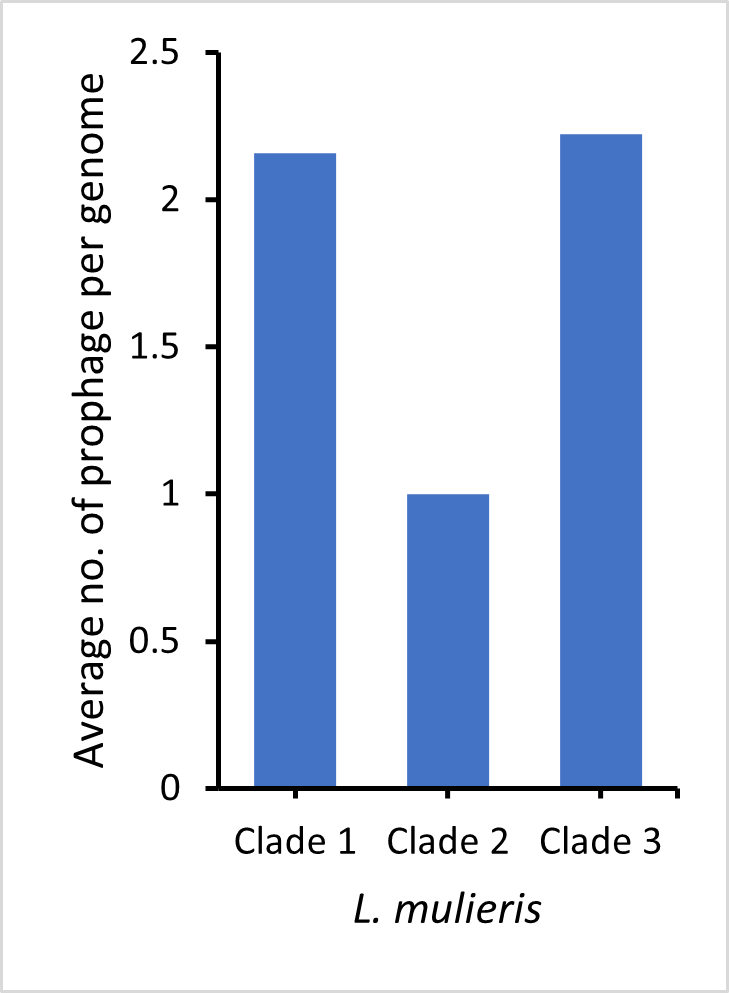


Fig. S1 Average number of prophages detected per genome across *L. mulieris* strains grouped by clade.
