# Supplementary material for: Pangenome analysis of Lactobacillus mulieris strains reveals distinct subspecies clusters with defined ecological adaptations
Source: Microbiol Spectr. 2025 Oct 2;13(11):e02011-25. doi: 10.1128/spectrum.02011-25 (PMC12584728; doi:10.1128/spectrum.02011-25)
Supplement: Supplemental methods — and references. [file spectrum.02011-25-s0004.docx]

**Supplementary Methods**

**Genome sequences**

The genome sequences used in this study were retrieved from the National Center for Biotechnology Information (NCBI) Datasets database (<https://www.ncbi.nlm.nih.gov/datasets/>) in September 2024. Initial identity verification of the genome sequences was assessed by cross-checking with the information available in the Genome Taxonomy Database (GTDB) (<https://gtdb.ecogenomic.org/>). Information of the genome sequences is listed in Table S1.The quality of the genome sequences was assessed using CheckM2 (1). Only the genome sequences with >90% completeness and <5% contamination were included in the analysis.

**Genome size and GC content analyses**

Genome size and GC content data for each genome sequences were obtained from the GTDB. Data were treated statistically using analysis of variance (ANOVA) with Tukey multiple-comparison post hoc test implemented in Microsoft Excel 2021. Graphical representations were generated using Microsoft Excel 2021 and Chiplot (<https://www.chiplot.online/>).

**Phylogenomic tree reconstruction**

Single-copy genes from all the genome sequences including the outgroups were identified using OrthoFinder version 3 (v.3) (2) using the default parameters. By default, OrthoFinder runs DIAMOND (3) in default mode, and to cluster genes into orthogroups, an mcl inflation parameter of 1.5 is used. The maximum likelihood tree was generated from the multiple sequence alignment using the same tool with the specific command ‘-M msa’. By default, OrthoFinder uses MAFFT (4) for the alignment and FastTree (5) for the tree inference. The resulting phylogenomic tree was visualized using Chiplot (<https://www.chiplot.online/>).

**Pangenome analysis**

Anvi’o version 8 (v.8) was used for the pangenome analysis applying the default parameters unless otherwise specified. Briefly, the following commands were executed: ‘anvi-gen-contigs-database’ was employed to convert each FASTA genome files into anvi’o contigs database. The ‘anvi-run-hmms’ and ‘anvi-run-ncbi-cogs’ were used to annotate the contigs database with functions from the NCBI’s clusters of orthologous groups (COG) database (6). The ‘anvi-gen-genomes-storage’ was employed to generate the anvi’o genomes storage. Using the generated anvi’o genomes storage, ‘anvi-pan-genome’ was used to conduct the actual pangenome analysis, with the flag ‘--use-ncbi-blast’, the parameter ‘--mcl-inflation 10’, and the default min-bit parameter of 0.5. The results were then visualized in an interface using ‘anvi-display-pan’. Functional enrichment analysis of gene clusters was performed using the anvi’o built-in program with the command ‘anvi-compute-functional-enrichment-in-pan’. This program identifies characteristic functions within defined genome groups, highlighting those enriched in a specific group but largely absent elsewhere. Details of the pipeline can be found at <https://merenlab.org/2016/11/08/pangenomics-v2/#functional-and-geometric-homogeneity-estimates-in-anvio>.

**Determination of genome relatedness** **indices**

Pairwise average nucleotide identity (ANI) values were determined using the command-line OrthoANIu tool of the EzBiocloud (7) with the command ‘java -jar OAU.jar -u /path/to/usearch -fd /path/to/fasta_files_directory -o /path/to/desired_output_file’. No user-defined parameters were applied. The digital DNA-DNA hybridization (dDDH) values were calculated using formula *d_4_* of the Genome-to-Genome Distance Calculator (GGDC) implemented in the Type Strain Genome Server (TYGS) online service (8). For this purpose, the FASTA genome sequence files were uploaded to the TYGS online portal (<https://tygs.dsmz.de/>) and the automated workflow provided by the service was then executed. No user-defined parameters are required.

References

1. Chklovski A, Parks DH, Woodcroft BJ, Tyson GW. 2023. CheckM2: a rapid, scalable and accurate tool for assessing microbial genome quality using machine learning. Nat Methods 2023 208 20:1203–1212.

2. Emms DM, Kelly S. 2019. OrthoFinder: Phylogenetic orthology inference for comparative genomics. Genome Biol 20:1–14.

3. Buchfink B, Xie C, Huson DH. 2014. Fast and sensitive protein alignment using DIAMOND. Nat Methods 12:59–60.

4. Katoh K, Misawa K, Kuma KI, Miyata T. 2002. MAFFT: a novel method for rapid multiple sequence alignment based on fast Fourier transform. Nucleic Acids Res 30:3059–3066.

5. Price MN, Dehal PS, Arkin AP. 2009. FastTree: Computing Large Minimum Evolution Trees with Profiles instead of a Distance Matrix. Mol Biol Evol 26:1641–1650.

6. Galperin MY, Vera Alvarez R, Karamycheva S, Makarova KS, Wolf YI, Landsman D, Koonin E V. 2025. COG database update 2024. Nucleic Acids Res 53:D356–D363.

7. Yoon SH, Ha S min, Lim J, Kwon S, Chun J. 2017. A large-scale evaluation of algorithms to calculate average nucleotide identity. Antonie van Leeuwenhoek, Int J Gen Mol Microbiol 110:1281–1286.

8. Meier-Kolthoff JP, Carbasse JS, Peinado-Olarte RL, Göker M. 2022. TYGS and LPSN: a database tandem for fast and reliable genome-based classification and nomenclature of prokaryotes. Nucleic Acids Res 50:D801–D807.
