# Supplementary material for: Pangenome analysis of Lactobacillus mulieris strains reveals distinct subspecies clusters with defined ecological adaptations
Source: Microbiol Spectr. 2025 Oct 2;13(11):e02011-25. doi: 10.1128/spectrum.02011-25 (PMC12584728; doi:10.1128/spectrum.02011-25)
Supplement: Table S1 — Genome sequence dataset information. [file spectrum.02011-25-s0005.docx]

Table S1. Genome sequence dataset information.

| *Lactobacillus mulieris* | Strain | Type | Isolation Source | Completeness (%) | Contamination (%) | Size (bp) | G+C (%) | Accession | Bioproject | Identifyer | Sequencing Technology | Assembly method |
| --- | --- | --- | --- | --- | --- | --- | --- | --- | --- | --- | --- | --- |
| Clade 1 | c10Ua161M | yes | urine | 99.99 | 0.06 | 1,661,396 | 34.23 | GCF_007095465.1 | PRJNA516335 | Lactobacillus | Illumina MiSeq | SPAdes v. 3.12.0 |
|  | c10Ua232AE | no | urine | 99.99 | 0.06 | 1,660,545 | 34.22 | GCA_007095435.1 | PRJNA516336 | Lactobacillus | Illumina MiSeq | SPAdes v. 3.12.0 |
|  | UMB9245 | no | urine | 99.92 | 0.15 | 1,723,383 | 34.18 | GCF_012102955.1 | PRJNA970254 | Genomic Catalog of Human Bladder Bacteria | Illumina NextSeq | SPAdes v. 3.13.0 |
|  | CM_D01_S772-bin_1 | no | ectocervical mucosa/vagina | 99.32 | 0.06 | 1,565,593 | 33.97 | GCF_022455405.1 | PRJNA799737 | human vaginal metagenome | Illumina HiSeq | SPAdes v. 3.13.032 |
|  | UMB8354 | no | urine | 99.9 | 0.08 | 1,604,117 | 33.94 | GCF_021495055.1 | PRJNA970254 | Genomic Catalog of Human Bladder Bacteria | Illumina NextSeq | SPAdes v. 3.15.2 |
|  | UMB1186 | no | urine | 99.89 | 0.2 | 1,696,445 | 34.16 | GCF_021495025.1 | PRJNA970254 | Genomic Catalog of Human Bladder Bacteria | Illumina NextSeq | SPAdes v. 3.15.2 |
|  | CM_F05_S806-bin_1 | no | ectocervical mucosa/vagina | 99.87 | 0.06 | 1,586,611 | 33.96 | GCF_022455095.1 | PRJNA799737 | human vaginal metagenome | Illumina HiSeq | SPAdes v. 3.13.032 |
|  | DZD_CM_20_S788-bin_1 | no | ectocervical mucosa/vagina | 99.87 | 0.04 | 1,645,517 | 33.86 | GCF_022454455.1 | PRJNA799737 | human vaginal metagenome | Illumina HiSeq | SPAdes v. 3.13.032 |
|  | DZD_CM_06_S774-bin_1 | no | ectocervical mucosa/vagina | 99.96 | 0.06 | 1,596,910 | 34.14 | GCA_022454635.1 | PRJNA799737 | human vaginal metagenome | Illumina HiSeq | SPAdes v. 3.13.032 |
|  | C0109C3 | no | vagina | 99.99 | 0.05 | 1,730,806 | 34.09 | GCF_027583865.1 | PRJNA798061 | Sexually Transmitted Infection Network Graph | Illumina HiSeq | SPAdes v. spades-3.14.1 |
|  | UMB0047 | no | urine | 99.99 | 0.09 | 1,742,939 | 34.25 | GCF_030218625.1 | PRJNA970254 | Genomic Catalog of Human Bladder Bacteria | Illumina NextSeq | SPAdes v. 3.15.2 |
|  | C0089B2 | no | vagina | 98.95 | 0.3 | 1,692,855 | 34.26 | GCF_027584155.1 | PRJNA798061 | Sexually Transmitted Infection Network Graph | Illumina HiSeq | SPAdes v. spades-3.14.1 |
|  | UMB9245C | no | urine | 99.93 | 0.13 | 1,728,037 | 34.24 | GCF_030226095.1 | PRJNA970254 | Genomic Catalog of Human Bladder Bacteria | Illumina NextSeq | SPAdes v. v3.14.1 |
|  | UMB0007LM | no | urine | 99.94 | 0.27 | 1,770,201 | 34.04 | GCF_026184155.1 | PRJNA316969 | Bacteria: Female Urinary Microbiota Genome Sequencing | Illumina NovaSeq | SPAdes v. 3.14.1 |
|  | C0090C3 | no | vagina | 99.97 | 0.07 | 1,762,973 | 34.32 | GCF_027584075.1 | PRJNA798061 | Sexually Transmitted Infection Network Graph | Illumina HiSeq | SPAdes v. spades-3.14.1 |
|  | C0022B5 | no | vagina | 99.95 | 0.18 | 1,783,158 | 34.16 | GCA_027158955.1 | PRJNA797778 | Vaginal Microbiome Research Consortium | Illumina HiSeq | SPAdes v. 3.14.1 |
|  | UMB0007B | no | urine | 99.87 | 1.03 | 1,791,729 | 34.2 | GCF_030218965.1 | PRJNA970254 | Genomic Catalog of Human Bladder Bacteria | Illumina NextSeq | SPAdes v. v3.14.1 |
|  | UMB9984 | no | urine | 99.94 | 0.94 | 1,688,615 | 34.3 | GCF_030212205.1 | PRJNA970254 | Genomic Catalog of Human Bladder Bacteria | Illumina NextSeq | SPAdes v. v3.14.1 |
|  | C0127B3 | no | vagina | 99.95 | 0.11 | 1,770,640 | 34.25 | GCF_027158665.1 | PRJNA797781 | Vaginal Microbiome Research Consortium | Illumina HiSeq | SPAdes v. 3.14.1 |
|  | UMB9245B | no | urine | 99.92 | 0.13 | 1,713,024 | 34.13 | GCF_030213925.1 | PRJNA970254 | Genomic Catalog of Human Bladder Bacteria | Illumina NextSeq | SPAdes v. v3.14.1 |
|  | UMB0021 | no | urine | 99.98 | 0.07 | 1,621,983 | 34.23 | GCF_026184175.1 | PRJNA970254 | Genomic Catalog of Human Bladder Bacteria | Illumina NextSeq | SPAdes v. 3.14.1 |
|  | C0127B5 | no | vagina | 99.93 | 0.19 | 1,783,646 | 34.24 | GCF_027157445.1 | PRJNA797778 | Vaginal Microbiome Research Consortium | Illumina HiSeq | SPAdes v. 3.14.1 |
|  | VSI18 | no | vagina | 97.5 | 1.33 | 1,813,263 | 34.45 | GCA_029011575.1 | PRJNA934404 | Human vaginal bacterial isolates genome sequencing and assembly | Oxford Nanopore MinION | trycycler v. v0.4.1 |
|  | TL2937 | no | healthy adult feces | 99.13 | 0.06 | 1,675,234 | 34.24 | GCF_001742045.1 | PRJNA339464 | Lactobacillus jensenii strain:TL2937 | Illumina MiSeq | NGEN (DNAStar) v. 12.2.0 |
|  | JV-V16 | no | Vagina | 99.98 | 0.08 | 1,604,632 | 34.39 | GCA_000159335.1 | PRJNA543187 | Development & evaluation of Morphoseq synthetic long read sequencing technology | 454 | Newbler Assembler v. 1.1.03.24 |
|  | 27-2-CHN | no | Vagina | 99.93 | 0.08 | 1,639,455 | 34.09 | GCA_000161895.2 | PRJNA37953 | Lactobacillus jensenii 27-2-CHN | 454 | Newbler v. 2.0.0-PostRelease-1/21/2009 |
|  | 115-3-CHN | no | vagina | 99.92 | 0.07 | 1,648,660 | 34.06 | GCA_000162435.1 | PRJNA38645 | Lactobacillus jensenii 115-3-CHN | 454 | n/a |
|  | UMB1355 | no | urine, catheter | 99.93 | 0.17 | 1,730,238 | 34.05 | GCA_007786095.1 | PRJNA316969 | Female Urinary Microbiota Genome Sequencing | Illumina MiSeq | SPAdes v. 3.13.0 |
|  | UMB8440 | no | urine, catheter | 99.98 | 0.13 | 1,657,751 | 34.24 | GCA_008726405.1 | PRJNA316969 | Female Urinary Microbiota Genome Sequencing | Illumina MiSeq | SPAdes v. 3.11.1 |
|  | UMB4707 | no | urine, catheter | 99.95 | 0.1 | 1,632,711 | 34.17 | GCA_008728065.1 | PRJNA316969 | Female Urinary Microbiota Genome Sequencing | Illumina MiSeq | SPAdes v. 3.11.1 |
|  | UMB639 | no | urine, catheter | 99.76 | 0.12 | 1,650,116 | 34.17 | GCA_008728115.1 | PRJNA316969 | Bacteria: Female Urinary Microbiota Genome Sequencing | Illumina MiSeq | SPAdes v. 3.11.1 |
|  | FDAARGOS_749 | no | n/a | 99.98 | 0.08 | 1,609,019 | 34.47 | GCA_009730255.1 | PRJNA231221 | FDA-ARGOS | Pacbio; Illumina | canu v. 1.4 |
|  | MHKL_MAG_00102 | no | ectocervical mucosa/vagina | 93.11 | 0.18 | 1,496,006 | 33.87 | GCF_022751765.1 | PRJNA799445 | human vaginal metagenome | Illumina HiSeq 2000 | SPAdes v. 3.13.032 |
|  | MHKL_MAG_00082 | no | ectocervical mucosa/vagina | 99.66 | 0.09 | 1,573,744 | 34.01 | GCF_022752135.1 | PRJNA799445 | human vaginal metagenome | Illumina HiSeq 2000 | SPAdes v. 3.13.032 |
|  | MHKL_MAG_00004 | no | ectocervical mucosa/vagina | 99.75 | 0.16 | 1,547,315 | 33.97 | GCF_022753705.1 | PRJNA799445 | human vaginal metagenome | Illumina HiSeq 2000 | SPAdes v. 3.13.032 |
|  | MHKL_MAG_00210 | no | ectocervical mucosa/vagina | 99.77 | 0.09 | 1,539,335 | 33.9 | GCF_022753825.1 | PRJNA799445 | human vaginal metagenome | Illumina HiSeq 2000 | SPAdes v. 3.13.032 |
|  | MHKL_MAG_00279 | no | ectocervical mucosa/vagina | 99.83 | 0.1 | 1,547,863 | 34 | GCF_022756825.1 | PRJNA799445 | human vaginal metagenome | Illumina HiSeq 2000 | SPAdes v. 3.13.032 |
|  | MHKL_MAG_00230 | no | ectocervical mucosa/vagina | 99.98 | 0.09 | 1,606,148 | 34.07 | GCF_022757825.1 | PRJNA799445 | human vaginal metagenome | Illumina HiSeq 2000 | SPAdes v. 3.13.032 |
|  | UMB0047 | no | urine, bladder catheter | 99.99 | 0.09 | 1,766,664 | 34.42 | GCF_030218625.1 | PRJNA970254 | Genomic Catalog of Human Bladder Bacteria | Illumina NextSeq | SPAdes v. v3.14.1 |
|  | UMB0021 | no | urine, bladder catheter | 99.98 | 0.16 | 1,704,592 | 34.41 | GCF_030218805.1 | PRJNA970254 | Genomic Catalog of Human Bladder Bacteria | Illumina NextSeq | SPAdes v. v3.14.1 |
|  | MGYG-HGUT-02313 | no | human gut | 99.13 | 0.06 | 1,675,234 | 34.24 | GCF_902385715.1 | PRJEB33885 | The Unified Human Gastrointestinal Genome catalogue | Illumina | n/a |
|  | IM11 | no | n/a | 99.9 | 0.1 | 1,614,827 | 34.02 | GCA_001012655.1 | PRJNA231005 | Lactobacillus jensenii | Illumina GAIIx | SOAPdenovo v. 2.04 |
|  | IM59 | no | n/a | 99.95 | 0.1 | 1,667,743 | 34.18 | GCA_001012675.1 | PRJNA231005 | Lactobacillus jensenii | Illumina GAIIx | SOAPdenovo v. 2.04 |
|  | IM1 | no | n/a | 99.53 | 0.29 | 1,541,635 | 34.05 | GCA_001012735.1 | PRJNA231005 | Lactobacillus jensenii | Illumina GAIIx | SOAPdenovo v. 2.04 |
|  | IM3 | no | n/a | 99.8 | 0.09 | 1,624,754 | 33.99 | GCA_001012745.1 | PRJNA231005 | Lactobacillus jensenii | Illumina GAIIx | SOAPdenovo v. 2.04 |
| Clade 2 | UMB7800 | no | urine | 99.96 | 0.36 | 1,694,839 | 34.13 | GCF_021495045.1 | PRJNA316969 | Female Urinary Microbiota Genome Sequencing | Illumina NextSeq | SPAdes v. 3.15.2 |
|  | UMB8026 | no | urine | 99.91 | 0.08 | 1,630,661 | 34.21 | GCF_026184095.1 | PRJNA316969 | Female Urinary Microbiota Genome Sequencing | Illumina NovaSeq | SPAdes v. 3.14.1 |
|  | UMB7784 | no | urine | 99.97 | 0.23 | 1,716,367 | 34.24 | GCF_030215185.1 | PRJNA970254 | Genomic Catalog of Human Bladder Bacteria | Illumina NextSeq | SPAdes v. v3.14.1 |
|  | MHKL_MAG_00238 | no | ectocervical mucosa/vagina | 91.63 | 2.13 | 1,527,447 | 33.96 | GCF_022757625.1 | PRJNA799762 | human vaginal metagenome | Illumina HiSeq | SPAdes v. 3.13.032 |
|  | UMB7783 | no | bladder urine, bladder catheter | 99.97 | 0.09 | 1,687,279 | 34.16 | GCF_030228195.1 | PRJNA970254 | Genomic Catalog of Human Bladder Bacteria | Illumina NextSeq | SPAdes v. v3.14.1 |
| Clade 3 | C0159B1 | no | vagina | 99.96 | 0.11 | 1,812,333 | 34.22 | GCF_027155335.1 | PRJNA797778 | Vaginal Microbiome Research Consortium | Illumina HiSeq | SPAdes v. 3.14.1 |
|  | C0172B3 | no | vagina | 99.98 | 0.08 | 1,845,621 | 34.2 | GCF_027153865.1 | PRJNA797778 | Vaginal Microbiome Research Consortium | Illumina HiSeq | SPAdes v. 3.14.1 |
|  | C0172B2 | no | vagina | 99.98 | 0.11 | 1,843,847 | 34.19 | GCF_027153895.1 | PRJNA797778 | Vaginal Microbiome Research Consortium | Illumina HiSeq | SPAdes v. 3.14.1 |
|  | C0093F8 | no | vagina | 99.97 | 0.1 | 1,829,902 | 34.15 | GCF_027584015.1 | PRJNA798061 | Sexually Transmitted Infection Network Graph | Illumina HiSeq | SPAdes v. spades-3.14.1 |
|  | C0149A3 | no | vagina | 99.98 | 0.12 | 1,796,787 | 34.09 | GCF_027156085.1 | PRJNA797778 | Vaginal Microbiome Research Consortium | Illumina HiSeq | SPAdes v. 3.14.1 |
|  | C0160B5 | no | vagina | 99.97 | 0.13 | 1,816,785 | 34.26 | GCF_027155305.1 | PRJNA797778 | Vaginal Microbiome Research Consortium | Illumina HiSeq | SPAdes v. 3.14.1 |
|  | C0172B4 | no | vagina | 99.97 | 0.08 | 1,847,214 | 34.19 | GCF_027153885.1 | PRJNA797778 | Vaginal Microbiome Research Consortium | Illumina HiSeq | SPAdes v. 3.14.1 |
|  | C0160B3 | no | vagina | 99.96 | 0.12 | 1,810,990 | 34.22 | GCF_027155225.1 | PRJNA797778 | Vaginal Microbiome Research Consortium | Illumina HiSeq | SPAdes v. 3.14.1 |
|  | C0149A5 | no | vagina | 99.96 | 0.12 | 1,796,524 | 34.08 | GCF_027156035.1 | PRJNA797778 | Vaginal Microbiome Research Consortium | Illumina HiSeq | SPAdes v. 3.14.1 |
|  | C0161C1 | no | vagina | 99.96 | 0.1 | 1,811,935 | 34.22 | GCF_027154705.1 | PRJNA797778 | Vaginal Microbiome Research Consortium | Illumina HiSeq | SPAdes v. 3.14.1 |
|  | C0150A1 | no | vagina | 99.98 | 0.11 | 1,788,836 | 34.09 | GCF_027156005.1 | PRJNA797778 | Vaginal Microbiome Research Consortium | Illumina HiSeq | SPAdes v. 3.14.1 |
|  | C0160B4 | no | vagina | 99.96 | 0.12 | 1,809,620 | 34.22 | GCF_027155165.1 | PRJNA797778 | Vaginal Microbiome Research Consortium | Illumina HiSeq | SPAdes v. 3.14.1 |
|  | C0149A4 | no | vagina | 99.98 | 0.12 | 1,797,055 | 34.08 | GCF_027156065.1 | PRJNA797778 | Vaginal Microbiome Research Consortium | Illumina HiSeq | SPAdes v. 3.14.1 |
|  | C0036B4 | no | vagina | 99.96 | 0.12 | 1,811,357 | 34.22 | GCF_027155555.1 | PRJNA797778 | Vaginal Microbiome Research Consortium | Illumina HiSeq | SPAdes v. 3.14.1 |
|  | C0159B2 | no | vagina | 99.96 | 0.12 | 1,810,938 | 34.22 | GCF_027155265.1 | PRJNA797778 | Vaginal Microbiome Research Consortium | Illumina HiSeq | SPAdes v. 3.14.1 |
|  | C0161C2 | no | vagina | 99.97 | 0.09 | 1,799,835 | 34.22 | GCF_027154965.1 | PRJNA797778 | Vaginal Microbiome Research Consortium | Illumina HiSeq | SPAdes v. 3.14.1 |
|  | C0036B1 | no | vagina | 99.96 | 0.11 | 1,811,376 | 34.22 | GCF_027155625.1 | PRJNA797778 | Vaginal Microbiome Research Consortium | Illumina HiSeq | SPAdes v. 3.14.1 |
|  | C0081E5 | no | vagina | 99.97 | 0.12 | 1,806,658 | 34.12 | GCF_027584235.1 | PRJNA798061 | Sexually Transmitted Infection Network Graph | Illumina HiSeq | SPAdes v. spades-3.14.1 |
